# Complement factor B regulates cellular senescence and is associated with poor prognosis in pancreatic cancer

**DOI:** 10.1007/s13402-021-00614-z

**Published:** 2021-06-01

**Authors:** Reiri Shimazaki, Shigetsugu Takano, Mamoru Satoh, Mamoru Takada, Yoji Miyahara, Kosuke Sasaki, Hideyuki Yoshitomi, Shingo Kagawa, Katsunori Furukawa, Tsukasa Takayashiki, Satoshi Kuboki, Kazuyuki Sogawa, Shinichiro Motohashi, Fumio Nomura, Masaru Miyazaki, Masayuki Ohtsuka

**Affiliations:** 1 Department of General Surgery, Graduate School of Medicine, Chiba University, 1-8-1, Inohana, Chuo-ku, Chiba-shi, Chiba, 260- 8677 Japan; 2 Division of Clinical Mass Spectrometry, Chiba University Hospital, Chiba, 260-8677 Japan; 3 Department of Biochemistry, School of Life and Environmental Science, Azabu University, 252-5201 Kanagawa, Japan; 4 Department of Medical Immunology, Graduate School of Medicine, Chiba University, 260-8677 Chiba, Japan

**Keywords:** Pancreatic ductal adenocarcinoma, Secreted protein, Complement factor B, Proliferation, Senescence, p21, Regulatory T-cells

## Abstract

**Background:**

The interplay between cancer cells and stromal components, including soluble mediators released from cancer cells, contributes to the progression of pancreatic ductal adenocarcinoma (PDAC). Here, we set out to identify key secreted proteins involved in PDAC progression.

**Methods:**

We performed secretome analyses of culture media of mouse pancreatic intraepithelial neoplasia (PanIN) and PDAC cells using Stable Isotope Labeling by Amino acid in Cell culture (SILAC) with click chemistry and liquid chromatography-mass spectrometry (LC-MS/MS). The results obtained were verified in primary PDAC tissue samples and cell line models.

**Results:**

Complement factor B (CFB) was identified as one of the robustly upregulated proteins, and found to exhibit elevated expression in PDAC cells compared to PanIN cells. Endogenous CFB knockdown by a specific siRNA dramatically decreased the proliferation of PDAC cells, PANC-1 and MIA PaCa-II. CFB knockdown induced increases in the number of senescence-associated-β-galactosidase (SA-β-gal) positive cells exhibiting p21 expression upregulation, which promotes cellular senescence with cyclinD1 accumulation. Furthermore, CFB knockdown facilitated downregulation of proliferating cell nuclear antigen and led to cell cycle arrest in the G1 phase in PDAC cells. Using immunohistochemistry, we found that high stromal CFB expression was associated with unfavorable clinical outcomes with hematogenous dissemination after surgery in human PDAC patients. Despite the presence of enriched CD8^+^ tumor infiltrating lymphocytes in the PDAC tumor microenvironments, patients with a high stromal CFB expression exhibited a significantly poorer prognosis compared to those with a low stromal CFB expression. Immunofluorescence staining revealed a correlation between stromal CFB expression in the tumor microenvironment and an enrichment of immunosuppressive regulatory T-cells (Tregs), myeloid-derived suppressor cells (MDSCs) and tumor-associated macrophages (TAMs). We also found that high stromal CFB expression showed a positive correlation with high CD8^+^/Foxp3^+^ Tregs populations in PDAC tissues.

**Conclusions:**

Our data indicate that CFB, a key secreted protein, promotes proliferation by preventing cellular senescence and is associated with immunological tumor promotion in PDAC. These findings suggest that CFB may be a potential target for the treatment of PDAC.

**Supplementary Information:**

The online version contains supplementary material available at 10.1007/s13402-021-00614-z.

## Introduction

Pancreatic ductal adenocarcinoma (PDAC) is notable for its profuse desmoplastic stroma comprising activated fibroblasts, leukocytes and extracellular matrix components [1, 2]. Studies utilizing in vitro assays and transplantation models have concluded that various stromal elements of the tumor microenvironment (TME) can enhance cancer cell proliferation and invasion [3–7]. Various stromal cells may also contribute to immunosuppression, further supporting tumor survival and growth. Together, these observations have led to the paradigm that tumor stroma functions to support and promote cancer growth [8]. Based on this paradigm, the concept of “anti-stromal” therapy has emerged as a promising, albeit unproven, therapeutic option. In contrast, Rim et al. reported that some stromal constituents may act to restrain, rather than promote tumor progression [9]. Although the role of tumor stroma in PDAC is still controversial, it has been reported that interactions between cancer cells and stroma components through the action of secreted proteins could play crucial roles in PDAC progression.

Stable Isotope Labeling by Amino acid in Cell culture (SILAC) with click chemistry is a comprehensive, quantitative and sensitive tool for the analysis of secreted proteins even in the presence of serum [10]. Secreted proteins are labeled with an azido-containing amino acid allowing their capture from complex mixtures through click chemistry, thereby circumventing the need for extensive peptide fractionation. In addition, concomitant stable isotope labeling allows relative protein quantification by mass spectrometry.

The complement system is a central part of both the innate and acquired immune systems that serve as a first line of defense against pathogens and stressed host cells [11]. Complement-related proteins perform several immune and nonimmune functions in both circulatory blood and peritumoral tissues by mediating cell-cell and cell-stroma interactions. Recent studies have shown that complement is not exclusive to liver-derived intravascular and extravascular systems, and that its components can be secreted locally by tissue-resident and infiltrating cells [12]. Specifically, it has been highlighted that activation of the complement cascade in the TME may enhance tumor growth via multiple mechanisms [13].

Herein, we performed comparative secretome analyses between mouse pancreatic intraepithelial neoplasia (PanIN) cells and PDAC cells using SILAC with click chemistry to identify key secreted proteins. We focused on the clinical and molecular features of one identified secreted protein, complement factor B (CFB), in PDAC progression. We found that CFB regulates proliferation to prevent cellular senescence in PDAC cells. Our data may facilitate the development of novel PDAC treatment options.

## Materials and methods

### Patients and human tissue samples

PDAC tissues were obtained from 113 consecutive patients who underwent surgical resection in the Department of General Surgery, Chiba University Hospital, Japan, from January 2010 to December 2014 (the follow-up period is at least 5 years). All patients were diagnosed with primary PDAC histologically, and TNM classification was performed according to the UICC 8th edition. The study protocol (protocol #2958) was approved the ethics committees of Chiba University, and written informed consent was obtained from each patient before surgery.

### Murine and human pancreatic cell lines and culture conditions

Mouse primary pancreatic cells were cultured and maintained as described previously [14]. Murine PanIN (KC) cells were isolated from a mouse at a PanIN stage (Pdx1-cre;LSL-Kras^G12D/+^) and PDAC (KPC) cell lines were established from the pancreas of a murine primary PDAC (Pdx1-cre;LSL-Kras^G12D/+^;p53^R172H/+^) provided by Dr. Sunil Hingorani [15]. The human PDAC cell lines BxPC-3, PANC-1, MIA PaCa-II, Capan-2, Capan-1, AsPC-1, CFPAC-1 and Hs766T were obtained from the American Type Culture Collection (ATCC, Manassas, VA, USA). BxPC-3, PANC-1 and MIA PaCa-II cells were cultured in Dulbecco’s Modified Eagle Medium (DMEM; Sigma Aldrich, St Louis, MO, USA) supplemented with 10 % fetal bovine serum (FBS) and antibiotics (1 % penicillin and streptomycin). CFPAC-1 and Capan-2 cells were cultured in Iscove’s Modified Dulbecco’s Medium (IMDM; Thermo Fisher Scientific, Waltham, MA, USA) supplemented with 10 % FBS and antibiotics, and AsPC-1 cells were cultured in RPMI-1640 medium (Thermo Fisher Scientific) supplemented with 10 % FBS and antibiotics.

### SILAC with click chemistry

For stable isotope labeling by amino acid in cell culture (SILAC) experiments, murine PanIN (KC) and PDAC (KPC) cells were maintained in SILAC medium comprising DMEM supplemented with 10 % dialyzed FBS, L-lysine and L-arginine or ^13^C_6_-lysine and ^13^C_6_^15^N_4_-arginine (isotopic) at a concentration of 0.1 g/L for light or heavy stable isotope labeling. Both light and heavy isotope-labeled cells, seeded in 10 cm culture dishes at 60–70 % confluency growing in light and heavy isotope medium, were incubated for 30 min in methionine-free medium to deplete endogenous methionine followed by incubation with azidohomoalanine (AHA). AHA concentration and incubation time were optimized to 0.1 µM and 12 h, respectively. Collected media were centrifuged (8 min at 5000 *g*), after which EDTA-free protease inhibitor was added and the mixture was frozen at − 80 °C. All assays were performed in independent biological duplicates with reversed SILAC labels. Newly synthesized proteins from concentrated media were enriched using a Click-iT Protein Enrichment Kit (Invitrogen C10416).

### Protein identification and quantification by LC-MS/MS analysis

 Enriched proteins were digested by trypsin. Digested peptides were fractionated [16] with Stage-Tip [17] simultaneously with desalting. Each fractionated peptide was injected into a trap column (C18, 0.3 × 5 mm; DIONEX, CA, USA) and an analytical column (C18, 0.075 × 120 mm; Nikkyo Technos, Tokyo, Japan), which was attached to Ultimate 3000 (DIONEX). The flow rate of the mobile phase was 300 nL/min. The solvent composition of the mobile phase was programmed to change in 180-min cycles with varying mixing ratios of solvent A (2 % v/v CH3CN and 0.1 % v/v HCOOH) to solvent B (90 % v/v CH3CN and 0.1 % v/v HCOOH). Purified peptides were introduced from HPLC to a LTQ-Orbitrap XL (Thermo Scientific, San Jose, CA, USA). One full scan cycle was applied (350–1200 m/z, resolution 60,000) followed by top three data-dependent collision induced dissociation (CID) MS/MS scans. Dynamic exclusion was applied as follows: 1 repeat count, 30 min repeat duration, 500 exclusion list sizes, and 180 s exclusion duration. Proteome Discoverer (version 1.3.0, Thermo Scientific, San Jose, CA, USA) was used to identify and quantify proteins from the mass, tandem mass spectra. Peptide mass data were matched by searching the UniProtKB mouse database (SwissProt 2017, 17,165 entries). Database search parameters were as follows: peptide mass tolerance, 2 ppm; fragment tolerance, 0.6 Da; enzyme was set to trypsin, allowing up to two missed cleavages; dynamic modifications, methionine oxidation, ^13^C_6_, ^15^N_4_-arginine and ^13^C_6_-lysine; static modifications, cysteine carbamidomethylation. The minimum criteria of protein identification were applied with Xcorr vs. charge state filter, and the false discovery rate (FDR) was set to < 1 %. The FDR was estimated by searching against a randomized decoy database created by the Proteome Discoverer 1.3.0 program supplied by Thermo Scientific.

###  Western blot analysis

Extracted proteins were separated by electrophoresis on 5-12.5 % XV PANTERA Gels (DRC, Tokyo, Japan) and transferred to membranes (PerkinElmer, Waltham, MA, USA). The membranes were blocked with 5 % skim milk diluted in 0.1 % Tris-buffed saline with Tween-20 (TBS-T) or PhosphoBLOCKER (Cell Biolabs, Inc., San Diego, CA, USA) at room temperature for 60 min. Next, the membranes were incubated with primary antibodies overnight at 4 °C, and incubated with secondary antibodies in blocking buffer. The membranes were subsequently incubated with enhanced chemiluminescence detection reagent (Amersham™ ECL™ Prime Western blotting detection reagent; GE Healthcare, Buckinghamshire, UK) and developed using a LAS-4000UV mini luminescent image analyzer (Fujifilm, Tokyo, Japan). The intensity of each band was quantified by densitometry using Image J software and used to calculate the relative protein level normalized to β-actin. A list of antibodies used is provided in Supplementary Information (Table S1).

### Short interfering RNA and reagents

A short interfering RNA (siRNA) that specifically targets human *CFB* mRNA (CFBsiRNA) to knockdown CFB expression was purchased from Ambion (Austin, TX, USA; CFBsiRNA; Cat # 4,427,037), and a control siRNA (si-control) was obtained from Qiagen (Hilden, Germany, All Stars negative control siRNA). Three thousand cells were transfected with siRNAs (3 nmol/L final concentration) in Opti-MEM I Reduced Serum Medium (Thermo Fisher Scientific, Grand Island, NY, USA) using Lipofectamine™ RNAiMAX Transfection Reagent (Invitrogen, Carlsbad, CA, USA). At 72 h after siRNA transfection, knockdown efficiency was evaluated by Western blot analysis. The cells were used for the following assays 24 h after transfection.

### Proliferation assay

Cell proliferation was evaluated using a Cell Counting Kit-8 (CCK-8) assay (Dojindo, Tokyo, Japan), according to the manufacturer’s protocol. The absorbance value was measured at 450 nm to determine cell viability using a 96-well plate reader (iMark™ Microplate Reader; Bio-Rad Laboratories).

### Annexin V-PI apoptosis assay

Apoptosis was evaluated using an Annexin V-FITC apoptosis detection kit (Nacalai Tesque, Kyoto, Japan) in conjunction with flow cytometry. Cells were treated for 12 h ,washed in cold PBS, resuspended in binding buffer, and stained with 5 µl FITC-labeled Annexin V and 5 µl propidium iodide (PI). As a positive control, Mitomycin C (5 µg/ml) (Nacalai Tesque, Kyoto, Japan) was used for the induction of early apoptotic cells. Cells were analyzed by flow cytometry using a CANTO II system (Beckton-Dickinson, CA, USA). All Data were analyzed using FlowJo v10.1r5 software (Ashland, OR, USA).

### SA-β-galactosidase staining

Cellular senescence was evaluated using a Cellular Senescence Detection Kit SPiDER-β-Gal (Dojindo, Kumamoto, Japan) in conjunction with flow cytometry. Bafilomycin A1 working solution was added, after which the cells were incubated for 1 h in a 5 % CO2 incubator. Next, SPiDER-β-Gal working solution was added, and the cells were incubated for another 30 min. Staining for senescence associated-β-galactosidase (SA-β-gal) was evaluated by fluorescence microscopy, and assessed by flow cytometry.

### Immunohistochemistry (IHC)

Formalin-fixed paraffin-embedded tissue samples were cut into 4-µm-thick slices and deparaffinized. Antigens were activated by autoclaving the tissue slides in citric acid buffer (0.01 mol/L, pH 6.0) at 120 °C for 10 min. The slides were blocked with hydrogen peroxide (H_2_O_2_) diluted to 3 % with methanol for 15 min to inactivate endogenous peroxidase. IHC was performed using the hyper-sensitive polymer method (Dako EnVision + kit; Glostrup, Denmark) for CFB according to the manufacturer’s protocol. After protein blocking, the slides were incubated with anti-CFB polyclonal antibody (1:1000 dilution; Proteintech, Rosemont, IL, USA) overnight at 4 °C. Counterstaining was performed with hematoxylin before dehydration, penetration and mounting. The staining intensities of CFB were evaluated independently by two investigators and a pathologist. The staining intensity of islets of Langerhans cells was used as an internal positive control. CFB staining scores were based on the extent and intensity of staining. Scores of the extent of staining were based on the percentage of positive stroma area (0–25 %, 1; 26–75 %, 2; > 75 %, 3). Scores of the intensity of staining were: no staining, 0; weak staining, 1; moderate staining, 2; strong staining, 3). Overall scores of each slide were calculated by the formula: overall score = extent score × intensity score. A score of < 2 was considered as Low expression and a score of 2 or more was considered as High expression. The staining patterns of CD8 or Foxp3^+^ Treg cells were scored by counting tumor stromal lymphocytes with positive staining in an average of 5 different high-power fields (×400) and classified as above (High) and below (Low) the median (CD8; range: 2–48, median: 16, average: 16, Foxp3^+^ Treg; range: 1–83, median: 13, average: 16). A list of antibodies used is provided in Supplementary Information (Table S1).

### Immunofluorescence staining

Antigens were activated and blocking was performed as indicated above. The primary and secondary antibodies used are listed in Supplementary Information. ProLong™ Gold antifade reagent with DAPI (Invitrogen) was used for counter staining of nuclei.

### Statistical analysis

Correlations between CFB expression and PDAC patient characteristics were evaluated by χ^2^ test, Mann-Whitney U test and Tukey’s HSD test. Survival rates were calculated using Kaplan-Meier analysis and assessed by log-rank test. Data were generated from *in vitro* experiments carried out at least three times independently, and analyzed by χ^2^ test, Tukey’s HSD test and multivariate analysis of variance (ANOVA). *P* values < 0.05 were considered to be statistically significant. Values are expressed as the mean ± standard error of the mean (SEM) or standard deviation (SD). The above statistical analyses were carried out using JMP® PRO 13 software (SAS Institute Inc., Cary, NC, USA).

## Results

### Identification by comprehensive secretome analysis of complement factor B as an upregulated secreted protein in pancreatic cancer

**Fig. 1:**
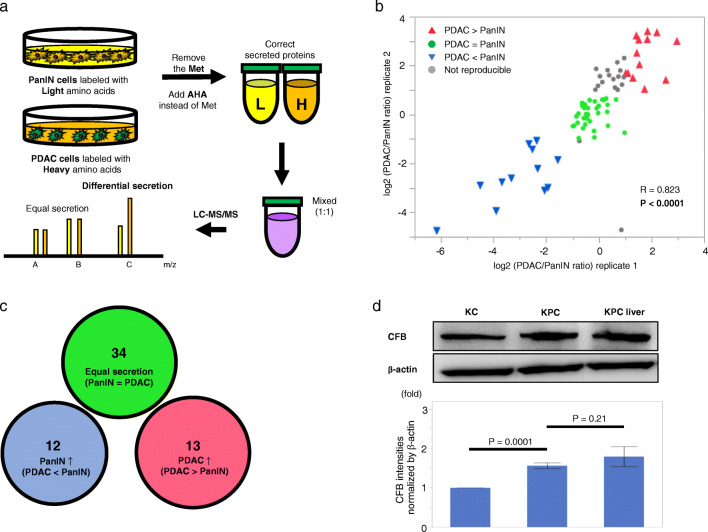
Comprehensive and comparative analyses of secreted proteins using SILAC with click chemistry and LC-MS/MS. **a** Experimental flow for identification of target proteins in this study. PanIN, pancreatic intraepithelial neoplasia; PDAC, pancreatic ductal adenocarcinoma; AHA, azidohomoalanine. **b** Validation of differential expression of 77 identified proteins in biological replicates in two independent experiments. **c** Validation of proteins using 2-fold constitutive change as a cutoff value for differential secretion. Twelve proteins with 2-fold higher secretion levels in PanIN cells (blue), 34 proteins with comparable secretion levels (green) and 13 proteins with 2-fold higher secretion levels in PDAC cells (red). **d** Comparative analysis of endogenous CFB protein expression levels in established mouse cell lines derived from (PanIN) cells (KC), PDAC in primary site (KPC), and its metastatic site in liver (KPCLiver) by Western blotting. Band intensities were normalized to β-actin. Experiments were performed in triplicates. Error bars represent standard deviation (SD)

To identify key secreted proteins involved in PDAC progression, comparative analyses of proteins present in the culture medium of mouse PanIN and PDAC cells were performed using SILAC with click chemistry and labeling by AHA (Fig. 1a). In total, 413 proteins were identified as secreted proteins by liquid chromatography/mass spectrometry (LC-MS/MS) (Table S2). Among them, 198 proteins were consistently identified and 77 proteins were found to exhibit highly correlated relative secretion levels between biological replicates (R = 0.823; *p* < 0.0001; Fig. 1b). By defining 2-fold constitutive change as a cutoff value of difference in secretion, we detected 13 proteins with higher secretion levels in PDAC cells (Fig. 1c). Among these 13 proteins (Table 1), we focused on complement factor B (CFB) for further analysis because it has recently been reported that activation of components of the complement cascade is closely associated with cancer progression [13]. We validated a stepwise upregulation in intrinsic cellular expression of CFB during PDAC progression (Fig. 1d).

**Table 1:** List of identified secreted proteins by SILAC and click-chemistry

Identified secreted proteins	Ratio (replicate 1)	Ratio (replicate 2)
DanJ homolog subfamily B member 9	10.5	3.61
4F2 cell-surface antigen heavy chain	10.2	4.63
Complement factor B	9.34	2.69
Mesothelin	8.40	3.59
Cadherin-1	8.30	2.86
Procollagen C-endopeptidase enhancer 1	7.80	7.83
Semaphorin-3 C	5.71	2.72
Extracellular matrix protein 1	4.10	2.92
Insulin-like growth factor-binding protein 3	3.22	2.15
Metalloproteinase inhibitor 1	3.21	2.03
Nidogen-1	2.81	2.46
Glyceraldehyde-3-phosphate dehydrogenase	2.65	5.86
Growth-regulated alpha protein	2.04	3.34

Ratio represents the ratio of secreted protein levels of PDAC cells to those of PanIN cells

### CFB is abundantly expressed in primary human PDAC tissues and cell lines

**Fig. 2:**
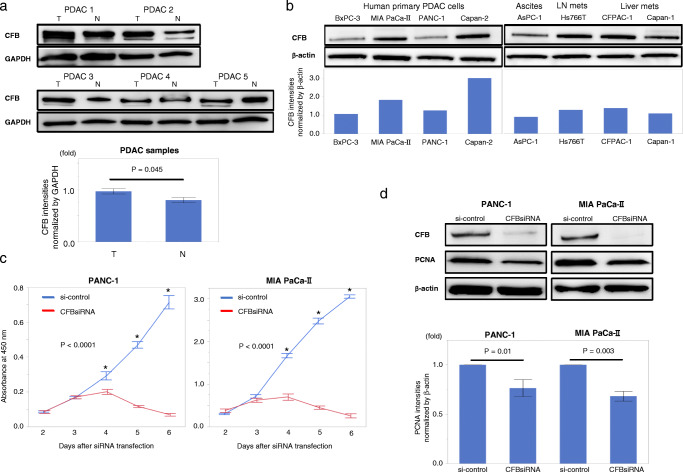
Differential expression of CFB in different human PDAC tissues and PDAC cell lines. **a** Upper panel: CFB protein expression in resected PDAC tissues by Western blot analysis. Lower panel: comparative analysis of CFB expression between tumor tissues and adjacent normal pancreatic tissues. Band intensities were normalized to GAPDH. Error bars represent SD. T: tumor tissues, N: adjacent normal pancreatic tissues. **b** Upper panels: CFB expression in various human PDAC cell lines from primary PDACs (BxPC-3, MIA PaCa-II, PANC-1, Capan-2), metastatic ascites (AsPC-1), lymph node metastasis (Hs766T) and PDAC liver metastases (CFPAC-1, Capan-1) detected by Western blot analysis. Lower panels: comparative analysis of CFB expression among PDAC cell lines. Band intensities were normalized to β-actin. **c** CFB knockdown impairs cell proliferation in PDAC cells. Significantly decreased proliferation in CFB-knockdown PANC-1 and MIA PaCa-II cells (*p* < 0.0001; MANOVA test). **d** Upper panel: PCNA expression evaluated in PANC-1 and MIA PaCa-II cells treated with si-control and CFBsiRNA by Western blotting. Lower panel: comparative analysis between CFB expression and PCNA expression. Band intensities were normalized to β-actin. Error bars represent SD

To confirm CFB expression in PDAC, its expression in human PDAC tissues and cell lines was assessed by Western blotting. We found that CFB was highly expressed in primary tumor samples compared to adjacent normal pancreatic tissues (Fig. 2a). CFB was also found to be expressed in all human PDAC cell lines tested (Fig. 2b). To next explore the relationship between CFB expression and epithelial plasticity in PDAC cells, endogenous CFB expression was suppressed by siRNA. We found that CFB knockdown altered neither E-cadherin nor vimentin expression in PANC-1 or MIA PaCa-II cells (Supplementary Fig. 1). This finding suggests that intrinsic CFB expression is not involved in epithelial plasticity in PDAC cells.

### CFB knockdown dramatically decreases proliferation of PDAC cells

Next, to assess the direct effect of CFB on PDAC cells, we determined whether CFB expression affects the proliferation of PDAC cells. Interestingly, we found that siRNA-mediated CFB knockdown resulted in dramatic decreases in the proliferation rates of both PANC-1 and MIA PaCa-II cells (*p* < 0.0001; Fig. 2c). Subsequently, we found that the expression of proliferating cell nuclear antigen (PCNA), which is upregulated during the S phase of cell cycle and plays a role in DNA replication, was also repressed in CFB-knockdown PDAC cells (Fig. 2d). These results indicate that CFB regulates PDAC cell proliferation via cell cycle control.

### Apoptosis is not involved in proliferation suppression of CFB knockdown PDAC cells

Apoptosis is one of the major phenomena of programmed cell death. Therefore, we first assessed whether apoptotic cell death is a canonical mechanism for inhibition of proliferation of CFB knockdown PDAC cells. We found that CFB knockdown did not increase the number of early apoptotic cells as demonstrated by annexin V/PI staining (Supplementary Fig. 2a-b). Additionally, we did not observe any increase in cleaved caspase-3 levels in CFB-knockdown cells (Supplementary Fig. 2c). These data indicate that the decreased cell proliferation observed in CFB knockdown PDAC cells is not likely to be due to apoptotic cell death.

### Loss of CFB induces senescence with p21 upregulation in PDAC cells

**Fig. 3:**
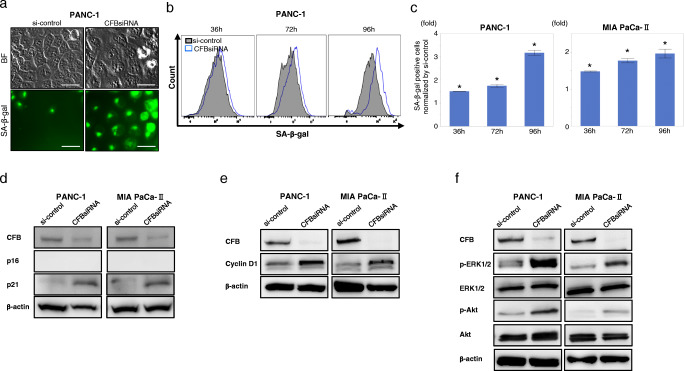
CFB knockdown by CFBsiRNA induces cellular senescence in CFB knockdown PDAC cells. **a** Representative image of SA-β-gal staining of PANC-1 and MIA PaCa-II cells treated with si-control or CFBsiRNA. Upper panels: bright field pictures; Lower panels: immunofluorescence pictures. **b**, **c** Analysis of SA-β-gal staining by flow cytometry in PDAC cells treated with si-control or CFBsiRNA. **b** Overlay of SA-β-gal positive populations of PANC-1 cells treated with si-control or CFBsiRNA. **c** Time-dependent analysis of SA-β-gal positive cells. *p < 0.0001 compared with si-control. **d** Western blot analysis of p16 and p21 expression in PDAC cells treated with si-control or CFBsiRNA. **e** Western blot analysis of cyclinD1 expression in PDAC cells treated with si-control or CFBsiRNA. **f** Western blot analysis for key mitogenic signaling pathways in CFB knockdown PDAC cells. p-ERK and p-Akt expression in PDAC cells treated with si-control or CFBsiRNA. Results are represented as mean ± SD. Each experiment was performed three times

Cellular senescence is characterized by an irreversible arrest in the G1 phase of the cell cycle, limiting the proliferation of primary human cells propagated *in vitro*. To explore whether intrinsic CFB expression is involved in the regulation of senescence, we performed SA-β-gal staining, one of the major hallmarks of cellular senescence. Remarkably, we found a significantly higher positive staining for SA-β-gal in CFB knockdown PDAC cells than in control cells (Fig. 3a). Also, a time-dependent increase in SA-β-gal positive staining was noted in cells treated with CFBsiRNA compared to si-control cells (Fig. 3b-c). We next assessed the expressions of pro-senescence factors, p21 and p16, in CFB-knockdown cells. Consistent with the above staining results, CFB knockdown caused an increase in p21 protein abundance in PDAC cells (Fig. 3d). To elucidate the mechanism underlying cell cycle arrest induction by senescence, we next assessed cyclin D1 expression (Fig. 3e). Interestingly, we found that increased cyclin D1 expression was accompanied by activation of upstream targets of key mitogenic pathways, i.e., phosphorylated extracellular signal-regulated kinase (ERK) [18] and phosphorylated protein kinase B (Akt) [19], in CFB knockdown PDAC cells (Fig. 3f, Supplementary Fig. 3). Taken together, these findings unveil a novel cell autonomous mechanism by which loss of endogenous CFB expression fosters p21-dependent cellular senescence in PDAC cells.

### High stromal CFB expression is associated with hematogenous recurrence and a poor prognosis after surgery in PDAC patients

**Fig. 4:**
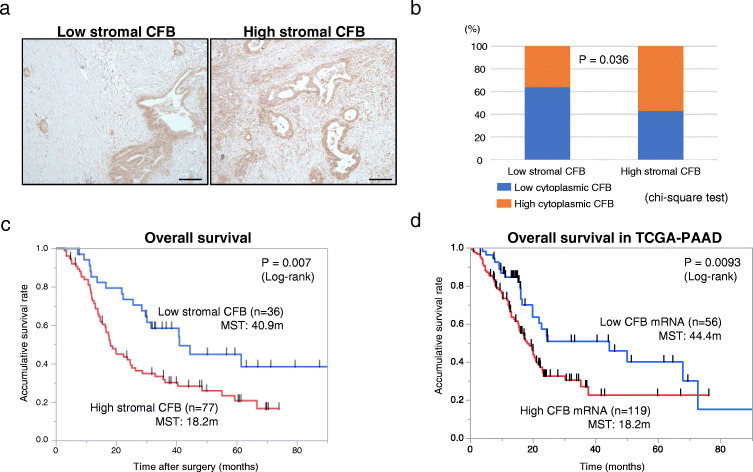
IHC analyses of CFB expression in resected human PDAC samples. **a** The staining patterns of CFB in primary PDAC tissue were categorized into low stromal CFB expression (left panel) or high stromal CFB expression (right panel) based on the intensity criterion (see Materials and Methods) (n = 113). Original magnification 200x. **b** Correlation of CFB expression between stroma and cytoplasm of cancer cells in human PDAC tissues. **c**, **d** Kaplan-Meier analyses of overall survival of patients with PDAC based on stromal CFB expression in the cohort of this study **c** and CFB mRNA expression in the TCGA-PAAD dataset **d**. **c** Patients in the high stromal CFB group presented significantly shorter overall survival times than patients in the low stromal CFB group after curative surgery (*p* = 0.007: log-rank test). **d** Patients in the high CFB mRNA group presented significantly shorter overall survival times than patients in the low CFB mRNA group in the TCGA-PAAD dataset (*p* = 0.0093: log-rank test). *P* values are indicated in the respective tables

To next investigate the clinical significance of CFB expression, we assessed CFB expression in 113 resected human PDAC samples by IHC staining. We found that CFB was primarily expressed in the stroma surrounding the tumor and in the cytoplasm of tumor cells (Fig. 4a). We measured CFB expression in both stroma and cytoplasm of tumor cells and found that among the 113 cases, 77 cases (68 %) were classified as high stromal CFB and 36 cases (32 %) as low stromal CFB, while 60 cases (53 %) were classified as high cytoplasmic CFB and 53 cases (47 %) as low cytoplasmic CFB. The respective staining patterns showed a positive correlation in PDAC tissues (*p* = 0.036; Fig. 4b). Considering the fact that CFB is secreted from PDAC cells, we focused on stromal CFB expression in evaluating clinical pathological features and outcomes. Interestingly, we found that the high stromal CFB group showed a significantly higher frequency of hematogenous recurrence (*p* = 0.0083; Table 2), and shorter disease free (*p* = 0.009; Supplementary Fig. 4) and overall survival (*p* = 0.007; Fig. 4c) compared to the low stromal CFB group. Upon multivariate analysis, we found that tumor size, venous invasion and stromal CFB expression served as independent markers for a poor prognosis of PDAC patients (Table 3). To validate these clinical data in an independent cohort, we evaluated CFB mRNA expression in a publicly available pancreatic ductal adenocarcinoma dataset of The Cancer Genome Atlas (TCGA-PAAD) [20]. The patients of the TCGA-PAAD cohort were divided into two groups based on the same percentage as in our cohort, (high CFB mRNA (119/175: 68 %) and low CFB mRNA (56/175: 32 %)). Similar to the above results, analysis of the TCGA-PAAD dataset revealed that the high CFB mRNA group again had a significantly worse prognosis compared to the low CFB mRNA group (*p* = 0.0093; Fig. 4d). These results implicate that high stromal CFB expression is associated with hematogenous recurrence and a poor prognosis in patients with PDAC after surgery.

**Table 2:** Correlation between stromal CFB expression and clinico-pathological features

	High stromal CFB (n = 77)	Low stromal CFB (n = 36)	*P* value
Sex (Male/Female)	44/33	20/16	0.87
Age (y.o. median: range)	67 (35–82)	65 (38–81)	0.25
Preoperative CA19-9 level (U/ml median: range)	245.5 (0.1–6530)	73.6 (0.1–6380)	0.36
NLR (median: range)	2.32 (0.86–7.60)	2.05 (0.93–5.75)	0.31
LMR (median: range)	3.75 (1.04–9.76)	4.85 (1.57–8.60)	0.087
PLR (median: range)	172 (42–942)	140 (82–325)	0.020*
Preoperative treatment (+/−)	36/41	15/21	0.61
Histological grade (poorly/others)	7/70	7/29	0.17
Tumor size (mm median: range)	32 (12–79)	30 (15–70)	0.26
Portal vein invasion (+/−)	39/37	14/22	0.22
Arterial invasion (+/−)	11/66	3/33	0.34
Lymphatic vessel invasion (0,1/2,3)	17/60	15/21	0.034*
Blood vessel invasion (0,1/2,3)	33/44	14/22	0.94
UICC-stage (I,II/III,IV)	33/44	27/9	0.0012*
T-stage (T1,2/T3,4)	52/25	30/6	0.07
N-stage (N0/N1,2)	19/58	12/24	0.34
M-stage (M0/M1)	71/6	35/1	0.17
Resectability (R0/R1,2)	34/43	11/25	0.16
Adjuvant chemotherapy	66/11	29/7	0.49
Local recurrence (+/−)	28/49	14/22	0.80
Hematogenous recurrence (+/−)	32/45	7/29	0.018*
Lymph node recurrence (+/−)	9/68	3/33	0.92

*: significant value. NLR: neutrophil to lymphocyte ratio. LMR: lymphocyte to monocyte ratio. PLR: platelet to lymphocyte ratio

**Table 3:** Univariate and multivariate analyses by Cox proportional hazard model

	Univariate analysis		Multivariate analysis	
	HR (95 % CI)	*P* value	HR (95 % CI)	*P* value
CA19-9 (U/ml) ( ≧ 250/< 250)	1.68 (1.05–2.67)	0.029	1.43 (0.87–2.37)	0.16
Tumor size (mm) ( ≧ 30/< 30)	1.83 (1.16–2.92)	0.010	1.65 (1.01–2.71)	0.047
ly (0,1/2,3)	0.47 (0.26–0.80)	0.004	0.98 (0.50–1.88)	0.96
v (0,1/2,3)	0.35 (0.22–0.56)	< 0.001	0.32 (0.18–0.57)	< 0.001
Lymph node metastasis (+/−)	2.04 (1.20–3.63)	0.007	1.35 (0.76–2.51)	0.32
Stromal CFB expression (high/low)	1.89 (1.15–3.87)	0.005	1.98 (1.17–3.47)	0.012

### Immunosuppressive cells accumulate in CFB-rich stroma in PDAC tissues

**Fig. 5:**
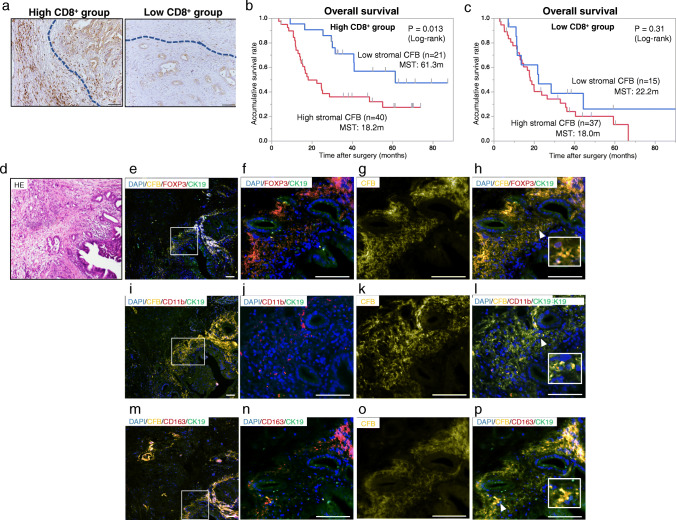
Correlations between immunocompetent or immunosuppressive cells and CFB expression in human PDAC tissues. **a** The staining patterns of CD8^+^ T cells in primary PDAC tissues were categorized into high CD8^+^ expression (upper panel) or low CD8^+^ expression (lower panel). The blue dotted line indicates the area of the invasive tumor front where CD8^+^ cells were counted. Original magnification 40x. **b**, **c** Kaplan-Meier analyses of overall survival of PDAC patients based on CFB expression in high and low CD8^+^ T-cell groups. **b** Patients with high stromal CFB presented a significantly shorter overall survival time (*p* = 0.013: log-rank test) than patients with low stromal CFB in the high CD8^+^ group. **c** Both patients with high and low stromal CFB showed a poor prognosis in the low CD8^+^ group. **d**-**p** Immunofluorescence analysis of immune cell markers, Foxp3 (Tregs), CD11b (MDSCs), and CD163 (TAMs) in human PDAC tissues. **d** Hematoxylin and eosin (H&E) staining. **e**-**h**, **i**-**l** and **m**-**p** Quadruple immunofluorescence staining for CK-19 (green), CFB (yellow), Foxp3/CD11b/CD163 (red) and DAPI (blue). Boxed regions highlight where stromal CFB is co-expressed with immunosuppressive cells, Tregs, MDSCs and TAMs in the TME of primary PDAC tissues. White arrowheads indicate representative regions where enrichment of immunosuppressive cells was observed in the CFB expressing stroma. Magnification 100x for (e, I, and m). Bar, 50 μm

We found that intrinsic and extrinsic CFB expression in PDAC tissues, and its stromal expression, were closely correlated with poor prognosis. Senescence is one of the physiological stress response programs characterized by loss of proliferative capacity, and it facilitates the recruitment of tumor infiltrating lymphocytes to the TME. Therefore, we hypothesized that the anti-senescence function of CFB may stimulate a pro-tumoral immune response by establishing an immunosuppressive TME. We assessed accumulation of tumor-infiltrating CD8^+^ T cells (CD8^+^ cells), which is one of the most crucial reactions of the host immune response against tumor cells. The number of CD8^+^ cells at the invasive front of the tumor was analyzed in resected human PDAC tissues by IHC (Fig. 5a). After division into high and low CD8^+^ groups, we found that the high CD8^+^ group showed a better prognosis compared to the low CD8^+^ group (*p* = 0.015; Supplementary Fig. 5a). Subsequent Kaplan-Meier analysis revealed that patients with low stromal CFB expression exhibited a significantly longer overall survival time than those with high stromal CFB expression in the high CD8^+^ group (*p* = 0.013; Fig. 5b). Of note, even in the high CD8^+^ group, the survival curve of the high stromal CFB group was almost equal to that of the low CD8^+^ group (Fig. 5b-c). These results were also validated through KM plotter analysis, which is an online database using gene expression data and survival information of cancer patients downloaded from the Gene Expression Omnibus (GEO), the European Genome-phenome Archive (EGA) and TCGA (Supplementary Fig. 5b-c) [21]. The data obtained suggest that high stromal CFB expression impacts the poor survival against T-cell mediated antitumor immunogenicity in PDAC patients.

**Fig. 6:**
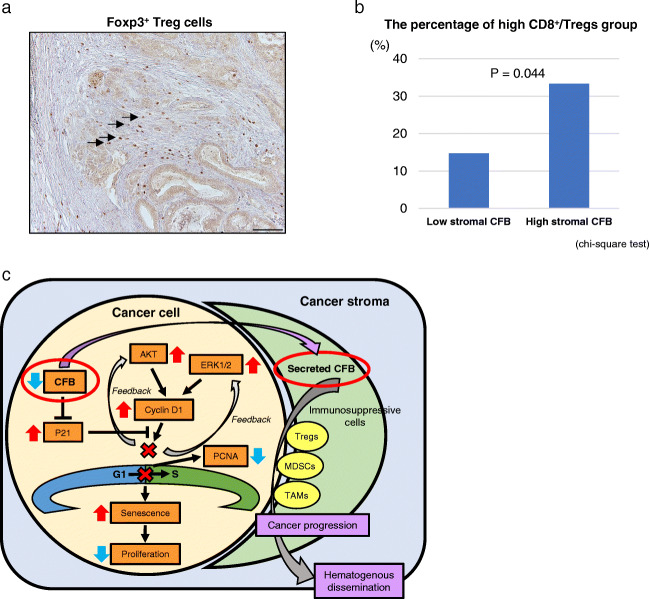
Correlation of CFB expression and the presence of CD8^+^/Foxp3^+^ Treg cells in resected PDAC tissues. **a** Black arrows show Foxp3^+^ Tregs in stroma surrounding pancreatic tumors. Original magnification 200x. **b** Correlation of CFB expression and CD8^+^/Foxp3^+^ Tregs in the TME of human PDAC tissues (*p* = 0.044, chi-square test). **c** Scheme of autonomous and non-autonomous CFB functions in PDAC cells and the tumor microenvironment

We finally examined correlations between CFB expression and the presence of pro-tumoral immune cells, promoting cancer cell spread and distant metastasis in human PDAC tissues. Immunofluorescence CFB staining and staining for immunosuppressive cell markers revealed their co-localization and dynamic enrichment of regulatory T cells (Tregs) (Fig. 5d-h), myeloid-derived suppressor cells (MDSCs) (Fig. 5i-l) and tumor-associated macrophages (TAMs) (Fig. 5m-p) in stroma surrounding the cancer cells in PDAC tissues. Recent research revealed that CD8^+^ Tregs accumulate in the TME and suppress antitumor immunity, thus promoting immune evasion and cancer progression [22, 23]. We assessed whether the high stromal CFB group is associated with the high CD8^+^/high Tregs PDAC group. The numbers of Foxp3^+^ cells surrounding the tumor were analyzed by IHC to divide the PDAC tissues into high and low Foxp3^+^ groups (Fig. 6a). We found that the percentage of high CD8^+^/Foxp3^+^ Tregs in the high stromal CFB group was significantly higher than that in the low stromal CFB group (*p* = 0.044; Fig. 6b). These data indicate that this phenotype is associated with immunological tumor promotion in the TME facilitating PDAC progression and imposing a poor clinical prognosis.

## Discussion

We identified CFB as an upregulated secreted protein in PDAC cells by comprehensive secretome analysis. Our *in vitro* data indicate that CFB plays a functional role in PDAC cancer cell survival by preventing cellular senescence via p21 expression regulation. IHC analyses revealed that high stromal CFB expression is associated with an unfavorable prognosis and correlates with recurrence and hematogenous dissemination after curative resection (Fig. 6c).

The central role of complement in inflammatory circuits is reflected by its involvement in acute and chronic inflammatory disorders with prominent complement imbalance [12]. It can be activated through three major pathways, i.e., the classic, lectin and alternative pathways [24]. Recent discoveries have revealed that complement effectors can also be generated intracellularly, which leads to its activation locally, independent of the plasmatic cascade [25]. CFB is known to play an important role in the alternative pathway of the complement cascade. CFB binds to C3(H_2_O) or C3b, and is cleaved by complement factor D into fragments Ba and Bb [26], which have a stimulatory effect on the proliferation of activated human B cells [27]. Complement functions vary across cancer types as it can promote or suppress tumor growth depending on the context. A previous report indicated that CFB is highly expressed in PDAC cells grown in conditioned media [28]. In this study, we demonstrated that the secreted CFB protein abundance in PDAC cells is higher than that in PanIN cells. IHC staining clearly showed high CFB expression in both cancer cells and stroma of resected human PDAC tissues. Notably, we found that stromal CFB expression in human PDAC was significantly associated with early recurrence with hematogenous dissemination. Taken together, these finding implicate that secreted CFB may contribute to PDAC progression.

Cell proliferation with an epithelial characteristic is one of the most fundamental traits for metastatic colonization [8]. Recent evidence strongly suggests that components of the complement cascade function in cancer cell proliferation and/or survival. In ovarian cancer cells complement activation products C3a, and subsequently C5a, activate their receptors, resulting in increased proliferation via a direct autocrine effect [29]. It has also been shown that CFB knockdown results in a significant decrease in proliferation in cutaneous squamous cell carcinoma [30]. Consistent with previous findings, we here report that attenuation of endogenous CFB expression decreases PDAC cell proliferation through a novel mechanism where loss of intrinsic CFB leads to cellular senescence by cell cycle arrest at the G1 phase. We also show that CFB knockdown induces senescence in a manner dependent on p21 instead of p16. Elevated cyclin D1 levels are universal markers of senescence. Indeed, despite the activation of mitogenic signaling pathways involving phosphorylation of ERK and Akt [31], we found that PDAC cells with suppressed CFB expression cannot activate cyclin D1 and fail to progress into the S phase. In terms of clinical application, therapy-induced senescence is a well-known response to cancer therapy which has long been considered to be related to a favorable outcome and to be a basis for the development of novel therapies that induce a cytostatic response [32]. Indeed, CDK4/6 inhibition is a hot topic and anticipated to be a therapeutic option for various cancers [31]. Conversely, Milanovic et al. recently suggested that senescence is associated with complex cellular reprogramming that may eventually promote cancer stemness and give rise to a more aggressive cancer phenotype [33]. Further experiments aimed at the regulation of senescence by CFB may underscore the context-dependent effects of senescence, including the potential involvement of the senescence-associated secretory phenotype (SASP) in cancer progression.

The TME is thought to highly affect cancer growth and spread, thereby impacting the clinical outcome of cancer patients. The majority of PDACs display highly immunosuppressive features with a TME rich in Tregs, MDSCs and TAMs, indicating “immuno-escape” for evading the host immune response [34]. Imbalanced activation of complement in the TME triggers the release of immunosuppressive cytokines, not only by cancer cells but also by Tregs and MDSCs which inhibit anti-tumor CD8^+^ T-cell responses. A recent study has provided evidence that local complement activation of C3a and C5a in the TME results in an immunosuppressive response to melanoma through the recruitment of MDSCs and inhibition of CD8^+^ tumor infiltrating lymphocyte function [35]. Since CFB plays a crucial role in activation of the C3-C5 cascade in the alternative pathway, we hypothesized that CFB may be governed by PDAC cancer cells and act on the immunosuppressive response orchestrated by Tregs, MDSCs and TAMs in the TME. In line with this idea, we found that CFB co-localizes with tumor infiltrating immunosuppressive cells, resulting in cancer progression despite the presence of CD8^+^ tumor infiltrating lymphocytes in the TME of PDAC tissues. We also found that high stromal CFB expression showed a positive correlation with high CD8^+^/Foxp3^+^ Treg populations in PDAC tissues. Very recently, Gadwa et al. reported that depletion of Tregs reversed tumor growth, and that combination of Treg depletion and C3a and C5a receptor inhibition decreased tumor growth in head and neck cancer [36]. These results implicate CFB as a putative therapeutic target combined with immunomodulation therapy. Collectively, our results suggest that CFB secreted from tumor cells leads to a bypass of senescence and fosters cooperation between tumor cells and pro-tumoral immune cells in favor of PDAC progression.

We also observed a significant correlation between CFB expression and hematogenous recurrence in resected PDAC tissue samples. Supporting this observation, a recent study indicated that a molecular signature comprising eight degradome-related genes including CFB can be used to discriminate patients at risk of distant metastasis [37]. Related to clinical application, a recent report described the identification of CFB as a novel serologic biomarker for PDAC diagnosis using an integrated proteomic analysis. CFB showed a distinctly higher specificity than CA19-9 for PDAC against other types of digestive cancers and in discriminating PDAC patients from non-PDAC patients [38]. Furthermore, consistent with our findings, Kim et al. recently demonstrated that high CFB expression in plasma of PDAC patients is highly associated with a poor prognosis with early recurrence after pancreatectomy [39]. Thus, CFB may serve not only as a therapeutic target, but also as a diagnostic and prognostic biomarker for PDAC.

In conclusion, we found that endogenous CFB regulates p21-dependent cellular senescence and that its stromal expression is associated with a poor prognosis in PDAC. These findings provide novel insight into a context-dependent role of complement in PDAC progression. A limitation of our current study is that the role of CFB was not investigated in an *in vivo* model. Further studies are warranted to examine the functional role of intrinsic and extrinsic CFB expression in PDAC progression, and to determine whether CFB may serve as a promising *in vivo* therapeutic target for PDAC.

## Supplementary Information

ESM 1 (DOCX 16 kb)

ESM 2 (XLSX 164 kb)

ESM 3 Supplementary Fig. 1 CFB knockdown in PANC-1 and MIA PaCa-II cells by CFBsiRNA was confirmed by western blot analysis. CFB and E-cadherin/vimentin expression by western blot in PANC-1 and MIA PaCa-II cells treated with the control siRNA (si-control) and CFBsiRNA as measured by western blot. Supplementary Fig. 2 Apoptosis is not involved in cell growth inhibition in CFB-knockdown PDAC cells. (a) PANC-1 and MIA PaCa-II were treated with si-control, CFBsiRNA, or mitomycin C (MitomyC) (5 μg/ml) as a positive control for apoptosis. Analysis of apoptosis by flow cytometry was assessed by Annexin V/PI double-staining. (b) Comparative analysis of subpopulation of early apoptotic cells in PDAC cells treated with si-control or CFBsiRNA. (c) Western blot analysis for caspase-3 and cleaved caspase-3 in PDAC cells treated with sicontrol or CFBsiRNA. MitomyC was used for the induction of apoptosis as a positive control. Results are represented as mean ± SD. Supplementary Fig. 3 Comparative analysis of p-ERK1/2 or p-Akt expression in PDAC cells treated with si-control or CFBsiRNA. The band intensities were normalized to that of ERK1/2 or Akt. Results are represented as mean ± SD. Supplementary Fig. 4 Kaplan-Meier analysis for disease free survival of patients with PDAC based on stromal CFB expression. Patients with high stromal CFB group presented significantly shorter disease free survival than patients with low stromal CFB group after curative surgery (*p* = 0.009: log-rank test). Supplementary Fig. 5 Kaplan-Meier analyses for overall survival of patients with PDAC based on CFB expression in CD8^+^ T-cell enriched and decreased group. (a) Patients with high CD8^+^ exhibited significantly better prognosis compared to those with low CD8^+^ in the cohort of this study (*p* = 0.015: log-rank test). (b, c) Kaplan-Meier analyses for overall survival of patients with high CFB and low CFB in high CD8^+^ group (b) and in low CD8^+^ group (c) using the KM plotter. (PPTX 7645 kb)

## Data Availability

All data generated or analyzed in this study are included in this article and its supplementary information files.

## Abbreviations

AHA: azidohomoalanine
Akt: protein kinase B
CFB: complement factor B
ERK: extracellular signal-regulated kinase
LC-MS/MS: liquid chromatography-mass spectrometry
MDSCs: myeloid-derived suppressor cells
PanIN: pancreatic intraepithelial neoplasia
PDAC: pancreatic ductal adenocarcinoma
SA-β-gal: senescence-associated-β-galactosidase
Tregs: regulatory T cells
SASP: senescence-associated secretory phenotype
SILAC: stable isotope labeling by amino acid in cell culture
siRNA: short interfering RNA
TAMs: tumor-associated macrophages
TME: tumor microenvironment
