# Supplementary material for: Complement factor B regulates cellular senescence and is associated with poor prognosis in pancreatic cancer
Source: Cell Oncol (Dordr). 2021 Jun 1;44(4):937–50. doi: 10.1007/s13402-021-00614-z (PMC8338870; doi:10.1007/s13402-021-00614-z)
Supplement: Supplementary file 1 — (DOCX 16 kb) [file 13402_2021_614_MOESM1_ESM.docx]

**Table S1. List of primary and secondary antibodies**

| **Primary antibody** | **Sources** | **Dilution** |
| --- | --- | --- |
| Anti-CFB polyclonal antibody | Proteintech, IL, USA | WB 1:5000  IHC 1:1000  IF 1:1000 |
| Anti-E-cadherin polyclonal antibody | Santa Cruze Biotechnology, Santa Cruz, CA | WB 1:1000 |
| Anti-Vimentin polyclonal antibody | Cell Signaling Technology, MA, USA | WB 1:2000 |
| Anti-PCNA monoclonal antibody | Santa Cruze Biotechnology, Santa Cruz, CA | WB 1:1000 |
| Anti-Caspase3 antibody | Santa Cruze Biotechnology, Santa Cruz, CA | WB 1:1000 |
| Anti-Cleaved caspase3 monoclonal antibody | Cell Signaling Technology, MA, USA | WB 1:1000 |
| Anti-CDKN2A/p16INK4a monoclonal antibody | Abcam, Cambridge, UK | WB 1:1000 |
| Anti-p21WAF1 monoclonal antibody | Calbiochem, CA, USA | WB 1:30 |
| Anti-Cyclin D1 monoclonal antibody | Abcam, Cambridge, UK | WB 1:500 |
| Anti-pERK1/2 monoclonal antibody | Cell Signaling Technology, MA, USA | WB 1:2000 |
| Anti-ERK1/2 monoclonal antibody | Cell Signaling Technology, MA, USA | WB 1:2000 |
| Anti-pAkt monoclonal antibody | Cell Signaling Technology, MA, USA | WB 1:2000 |
| Anti-Akt monoclonal antibody | Cell Signaling Technology, MA, USA | WB 1:2000 |
| Anti-CD8 alpha polyclonal antibody | Abcam, Cambridge, UK | IHC 1:200 |
| Anti-Foxp3 monoclonal Ab | eBioscience, CA, USA | IHC 1:50  IF 1:1000 |
| Anti-CD11b monoclonal antibody | Abcam, Cambridge, UK | IF 1:1000 |
| Anti-CD163 polyclonal antibody | R&D Systems, Inc. MN, USA | IF 1:1000 |
| Anti-Cytokeratin 19 monoclonal antibody | Dako, Glostrup, Denmark | IF 1:1000 |
| Anti-βactin monoclonal antibody | Cell Signaling Technology, MA, USA | WB 1:2000 |
| Anti-GAPDH polyclonal antibody | GeneTex Inc, CA, USA | WB 1:2000 |

| **Secondary antibody** | **Sources** | **Dilution** |
| --- | --- | --- |
| Anti-rabbit IgG horseradish peroxidase antibody | Santa Cruze Biotechnology, Santa Cruz, CA | WB 1:2000 |
| Anti-mouse IgGκ BP-HRP antibody | Santa Cruze Biotechnology, Santa Cruz, CA | WB 1:2000 |
| Alexa Fluor 488 donkey anti-rabbit IgG antibody | Life Technologies, CA, USA | IF 1:500 |
| Alexa Fluor 546 goat anti-rat IgG antibody | Invitrogen, CA, USA | IF 1:500 |
| Alexa Fluor 568 goat anti-mouse IgG antibody | Life Technologies, CA, USA | IF 1:500 |
| Alexa Fluor 633 donkey anti-goat IgG antibody | Invitrogen, CA, USA | IF 1:500 |
| Alexa Fluor 546 goat anti-rat IgG antibody | Invitrogen, CA, USA | IF 1:500 |

WB: western blotting, IHC: Immunohistochemistry, IF: Immunofluorescence staining
