# Supplementary material for: Complement factor B regulates cellular senescence and is associated with poor prognosis in pancreatic cancer
Source: Cell Oncol (Dordr). 2021 Jun 1;44(4):937–50. doi: 10.1007/s13402-021-00614-z (PMC8338870; doi:10.1007/s13402-021-00614-z)
Supplement: Supplementary file 3 — Supplementary Fig. 1 CFB knockdown in PANC-1 and MIA PaCa-II cells by CFBsiRNA was confirmed by western blot analysis. CFB and E-cadherin/vimentin expression by western blot in PANC-1 and MIA PaCa-II cells treated with the control siRNA (si-control) and CFBsiRNA as measured by western blot. Supplementary Fig. 2 Apoptosis is not involved in cell growth inhibition in CFB-knockdown PDAC cells. (a) PANC-1 and MIA PaCa-II were treated with si-control, CFBsiRNA, or mitomycin C (MitomyC) (5 μg/ml) as a positive control for apoptosis. Analysis of apoptosis by flow cytometry was assessed by Annexin V/PI double-staining. (b) Comparative analysis of subpopulation of early apoptotic cells in PDAC cells treated with si-control or CFBsiRNA. (c) Western blot analysis for caspase-3 and cleaved caspase-3 in PDAC cells treated with sicontrol or CFBsiRNA. MitomyC was used for the induction of apoptosis as a positive control. Results are represented as mean ± SD. Supplementary Fig. 3 Comparative analysis of p-ERK1/2 or p-Akt expression in PDAC cells treated with si-control or CFBsiRNA. The band intensities were normalized to that of ERK1/2 or Akt. Results are represented as mean ± SD. Supplementary Fig. 4 Kaplan-Meier analysis for disease free survival of patients with PDAC based on stromal CFB expression. Patients with high stromal CFB group presented significantly shorter disease free survival than patients with low stromal CFB group after curative surgery (p = 0.009: log-rank test). Supplementary Fig. 5 Kaplan-Meier analyses for overall survival of patients with PDAC based on CFB expression in CD8+ T-cell enriched and decreased group. (a) Patients with high CD8+ exhibited significantly better prognosis compared to those with low CD8+ in the cohort of this study (p = 0.015: log-rank test). (b, c) Kaplan-Meier analyses for overall survival of patients with high CFB and low CFB in high CD8+ group (b) and in low CD8+ group (c) using the KM plotter. (PPTX 7645 kb) [file 13402_2021_614_MOESM3_ESM.pptx]

## Slide 1
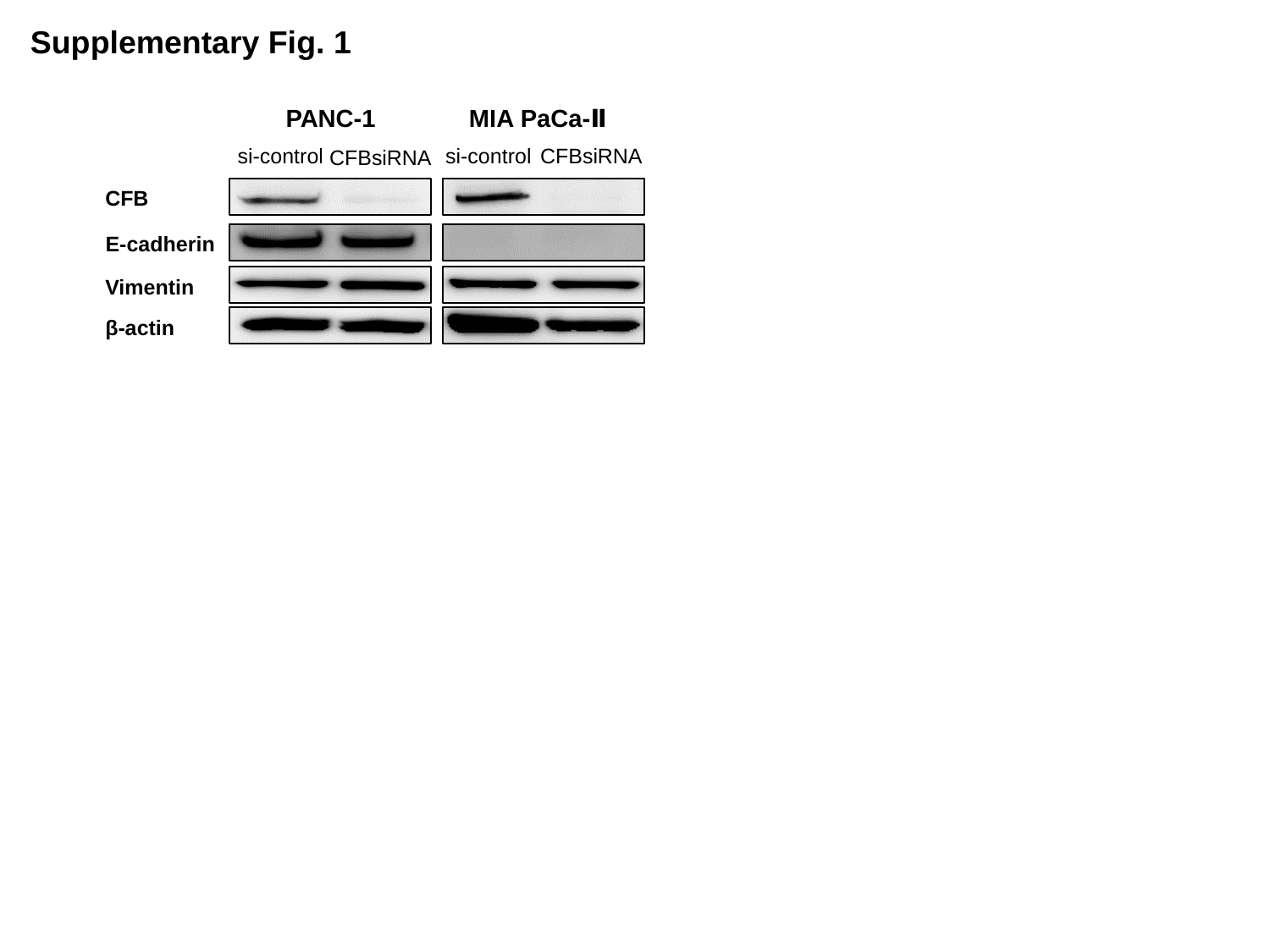

Supplementary Fig. 1
PANC-1
MIA PaCa-Ⅱ
si-control
si-control
CFBsiRNA
CFBsiRNA
CFB
E-cadherin
Vimentin
β-actin

## Slide 2
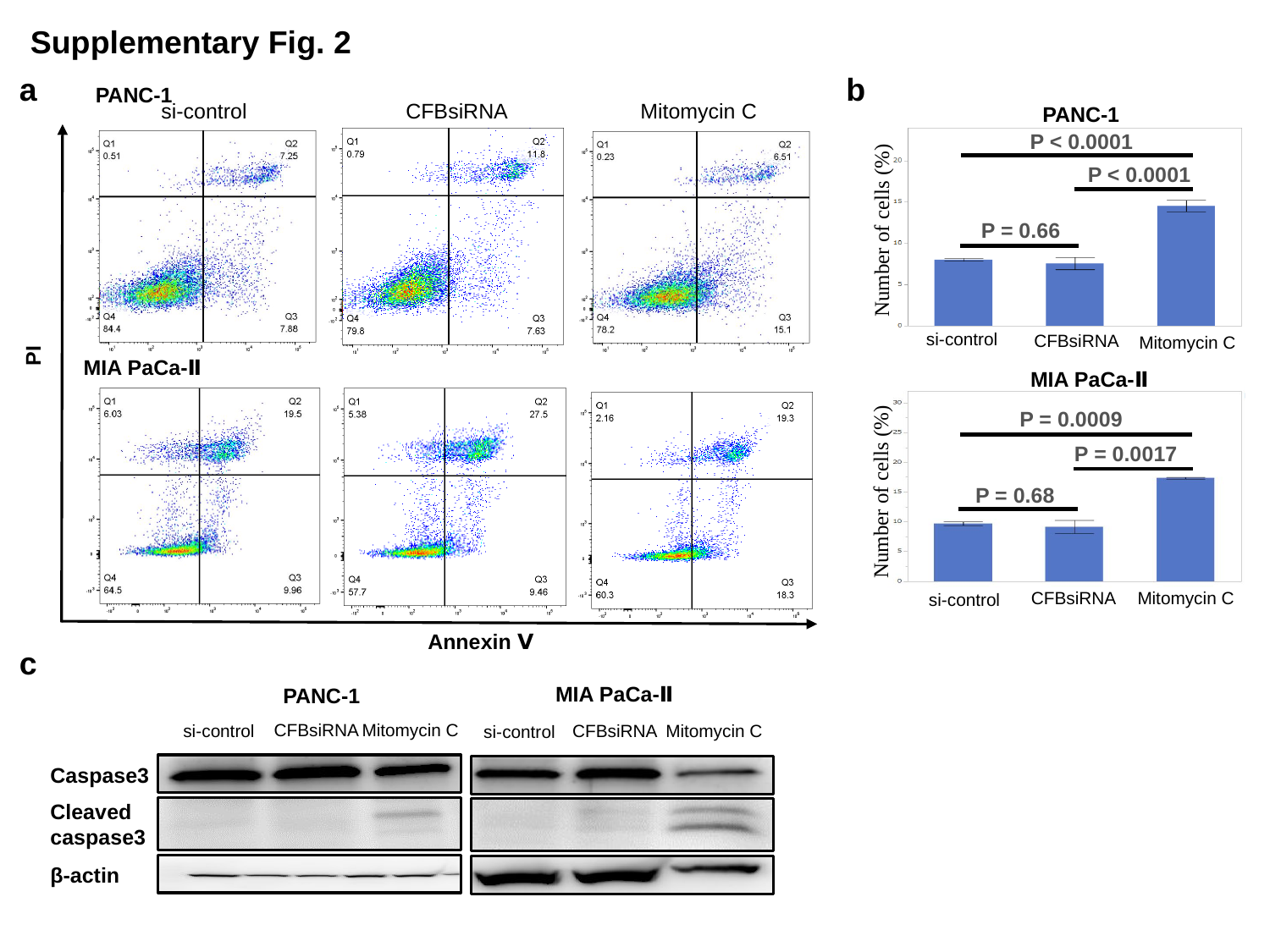

Supplementary Fig. 2
a
b
PANC-1
si-control
CFBsiRNA
Mitomycin C
PANC-1
P < 0.0001
P < 0.0001
P = 0.66
Number of cells (%)
si-control
CFBsiRNA
Mitomycin C
PI
MIA PaCa-Ⅱ
MIA PaCa-Ⅱ
P = 0.0009
P = 0.0017
Number of cells (%)
P = 0.68
CFBsiRNA
Mitomycin C
si-control
Annexin Ⅴ
c
MIA PaCa-Ⅱ
PANC-1
si-control
CFBsiRNA
Mitomycin C
si-control
CFBsiRNA
Mitomycin C
Caspase3
Cleaved caspase3
β-actin

## Slide 3
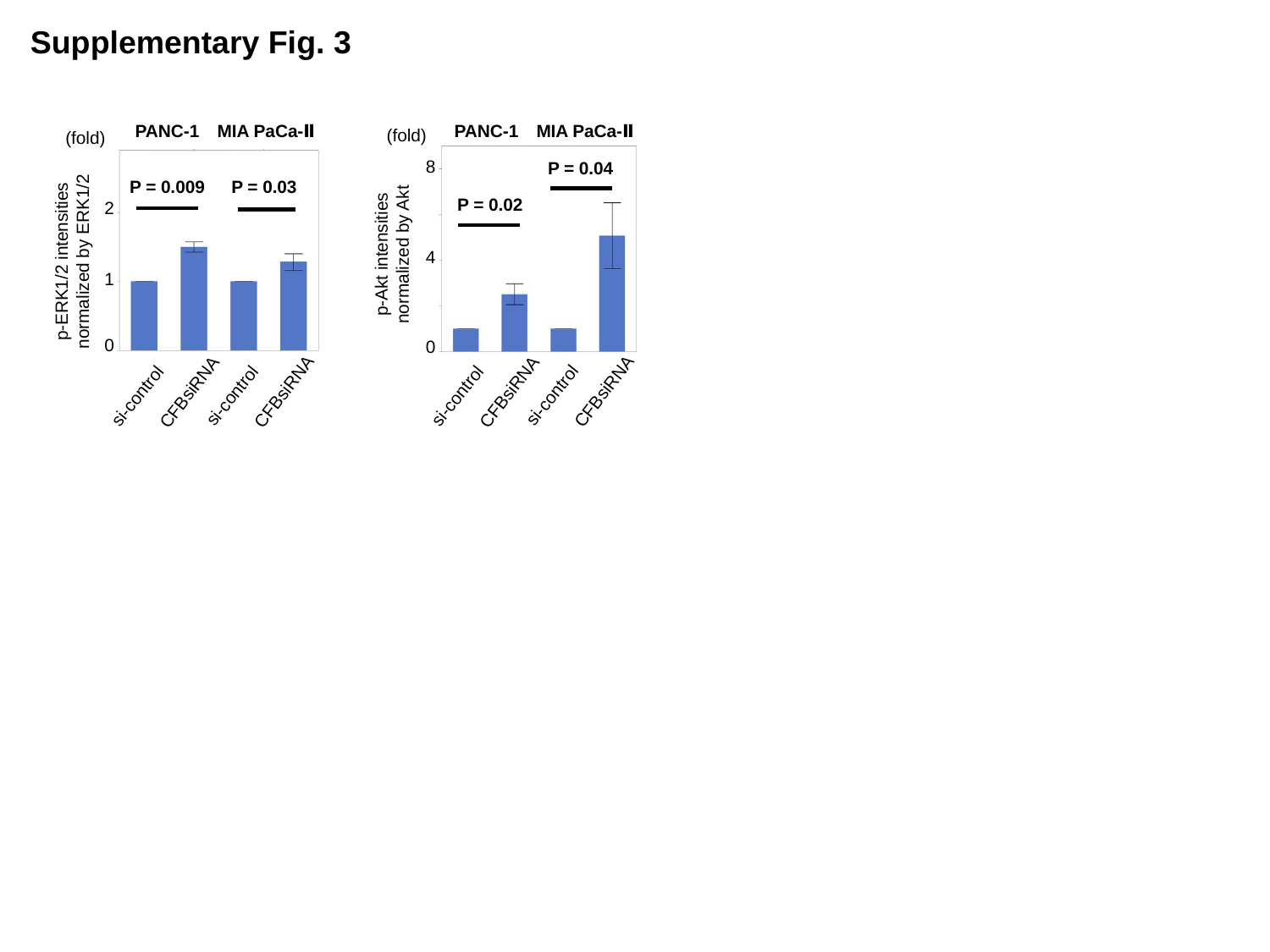

Supplementary Fig. 3
PANC-1
MIA PaCa-Ⅱ
PANC-1
MIA PaCa-Ⅱ
(fold)
8
P = 0.04
P = 0.02
p-Akt intensities
normalized by Akt
4
0
(fold)
P = 0.009
P = 0.03
2
p-ERK1/2 intensities
normalized by ERK1/2
1
0
CFBsiRNA
CFBsiRNA
CFBsiRNA
CFBsiRNA
si-control
si-control
si-control
si-control

## Slide 4
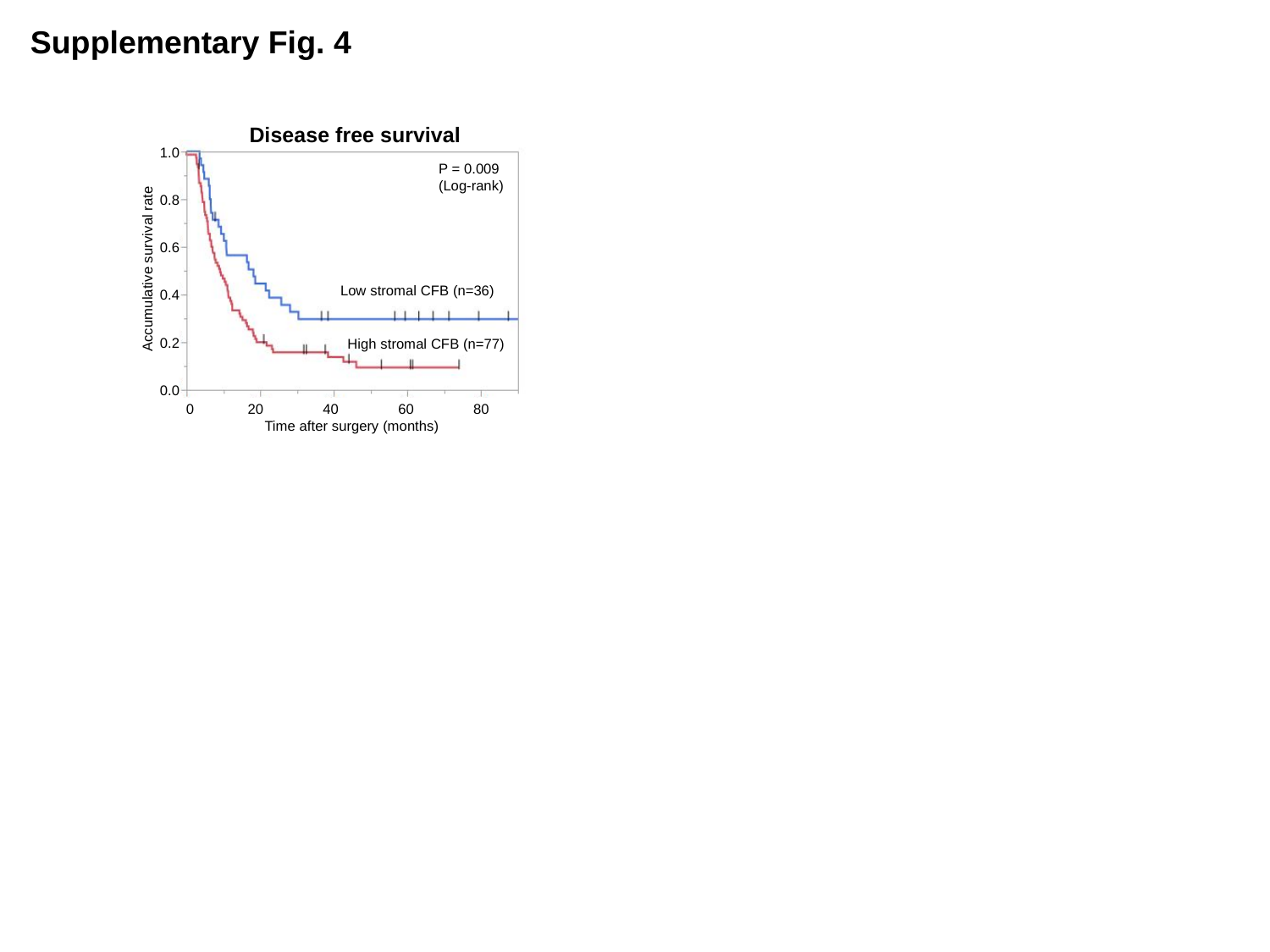

Supplementary Fig. 4
Disease free survival
1.0
P = 0.009
(Log-rank)
0.8
0.6
Accumulative survival rate
Low stromal CFB (n=36)
0.4
0.2
High stromal CFB (n=77)
0.0
0
20
40
60
80
Time after surgery (months)

## Slide 5
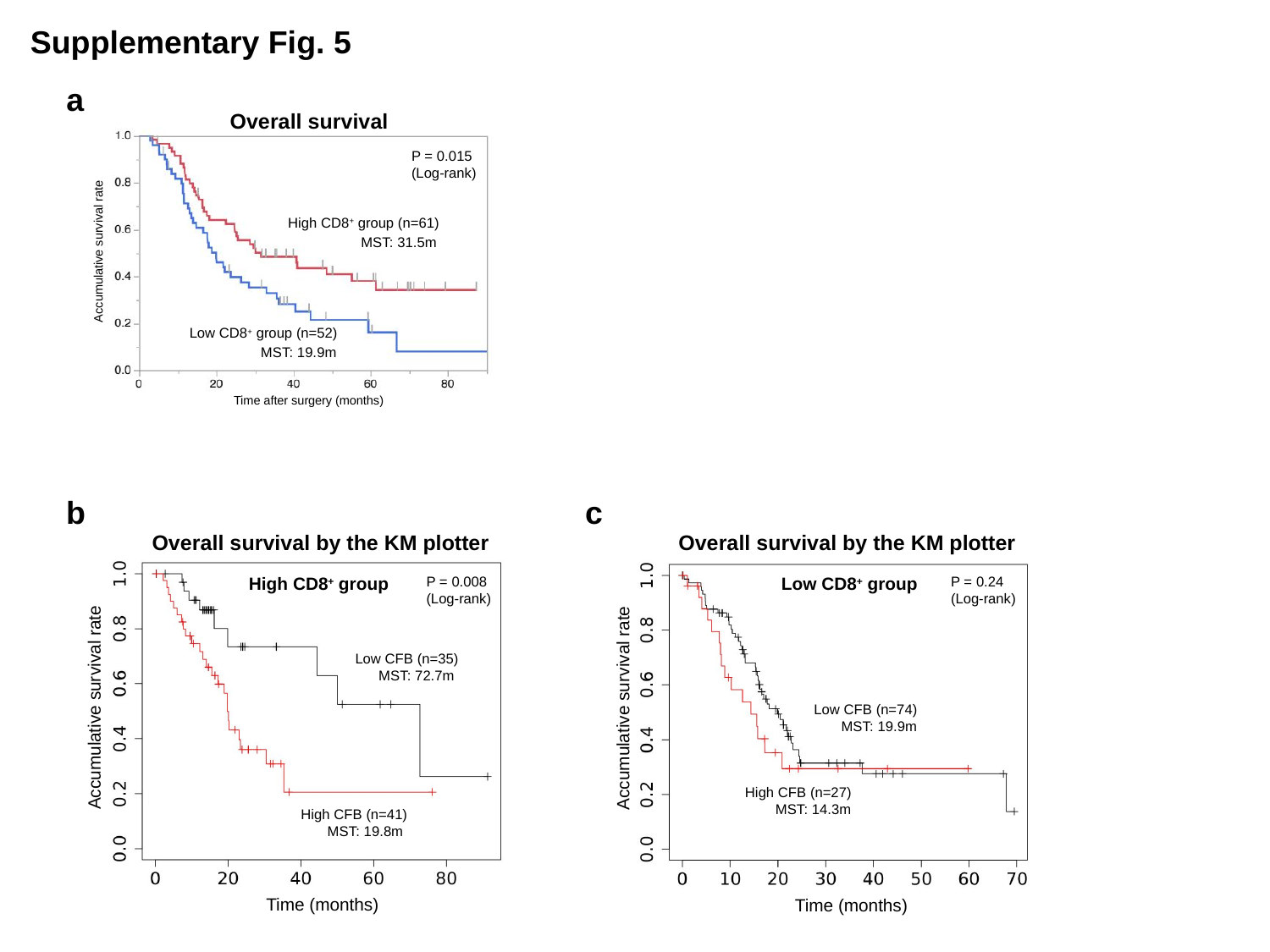

Supplementary Fig. 5
a
Overall survival
P = 0.015
(Log-rank)
High CD8+ group (n=61)
MST: 31.5m
Accumulative survival rate
Low CD8+ group (n=52)
MST: 19.9m
Time after surgery (months)
b
c
Overall survival by the KM plotter
Low CD8+ group
P = 0.24
(Log-rank)
Accumulative survival rate
Low CFB (n=74)
MST: 19.9m
High CFB (n=27)
MST: 14.3m
Time (months)
Overall survival by the KM plotter
P = 0.008
(Log-rank)
High CD8+ group
Low CFB (n=35)
MST: 72.7m
Accumulative survival rate
High CFB (n=41)
MST: 19.8m
Time (months)
